# Comparative transcriptome analysis reveals that ATP synthases regulate *Fusarium oxysporum* virulence by modulating sugar transporter gene expressions in tobacco

**DOI:** 10.3389/fpls.2022.978951

**Published:** 2022-08-18

**Authors:** Xiaotong Gai, Shuang Li, Ning Jiang, Qian Sun, Yuan Hu Xuan, Zhenyuan Xia

**Affiliations:** ^1^Research Center, Yunnan Academy of Tobacco Agricultural Sciences, Kunming, China; ^2^College of Life Science, Yan’an University, Yan’an, China; ^3^College of Plant Protection, Shenyang Agricultural University, Shenyang, China

**Keywords:** *Fusarium oxysporum*, virulence, comparative transcriptome, ATP synthase, sugar transporters, tobacco

## Abstract

*Fusarium oxysporum* is a main causative agent of tobacco root rot, severely affecting tobacco growth. Here, 200 *F. oxysporum* strains were isolated and examined for their virulence toward tobacco plants. These strains were divided into disease class 1–3 (weak virulence), 4–6 (moderate virulence), and 7–9 (strong virulence). To understand the virulence mechanism of *F. oxysporum*, a comparative transcriptome study was performed using weak, moderate, and strong virulence-inducing strains. The results showed that expression levels of 1,678 tobacco genes were positively correlated with virulence levels, while expression levels of 3,558 genes were negatively associated with virulence levels. Interestingly, the expression levels of ATP synthase genes were positively correlated with *F. oxysporum* virulence. To verify whether ATP synthase gene expression is associated with *F. oxysporum* virulence, 5 strains each of strong, moderate, and weak virulence-inducing strains were tested using qRT-PCR. The results confirmed that ATP synthase gene expression is positively correlated with virulence levels. Knock-out mutants of ATP synthase genes resulted in a relatively weak virulence compared to wild-type as well as the inhibition of *F. oxysporum*-mediated suppression of *NtSUC4*, *NtSTP12*, *NtHEX6*, and *NtSWEET*, suggesting that ATP synthase activity is also associated with the virulence. Taken together, our analyses show that ATP synthases are key genes for the regulation of *F. oxysporum* virulence and provide important information for understanding the virulence mechanism of *F. oxysporum* in tobacco root rot.

## Introduction

Tobacco root rot is a soil-borne disease caused by *Fusarium oxysporum* (Jie and Jing, 2013; Qiu et al., 2021a,b), that severely affects the production of tobacco. In Yunnan Province, the main tobacco cultivating area in China, root rot disease has greatly increased in severity in recent years. As the one of main tobacco root rot pathogen, *F. oxysporum* is a typical soilborne fungus that can colonize the plant vascular system and cause serious crop wilt disease (de Sain and Rep, 2015). The species affects more than 120 plant species, including some important crops such as cotton, tomato, melon, soybean as well as tobacco (Jie and Jing, 2013; de Sain and Rep, 2015; Edel-Hermann and Lecomte, 2019; Xu et al., 2020). Previous studies have shown that *F. oxysporum* infection suppressed the expression of sugar transporter genes including SUC, STP, HEX, and SWEET family members in tobacco, and *NtSWEET1* knock-down increased susceptibility of tobacco to *F. oxysporum* (Gai et al., 2021); however, the molecular mechanism of how tobacco plants defend themselves against *F. oxysporum* remains largely unknown.

On the other hand, *F. oxysporum* infects a broad range of hosts and in some of these its virulence mechanism has been analyzed. One study has found, that FoEG1, a protein secreted from *F. oxysporum*, modulates plant immunity and acts as a pathogen-associated molecular pattern (PAMP) targeting the apoplast of plants to induce cell death (Zhang et al., 2021). Galactofuranose-containing sugar chains contribute to the hyphal growth, conidiation, and virulence of *F. oxysporum* f. sp. cucumerinum (Zhou et al., 2021). It has been shown, that FocSge1 is required for the full virulence of *F. oxysporum* f. sp. cubense race 1 (Gurdaswani et al., 2020) while FoRlm1 regulates anti-oxidant enzymes to control aerial hyphal growth, oxidative stress, cell wall biosynthesis, and virulence in *F. oxysporum* f. sp. cubense (Ding et al., 2020). Moreover, SIX8 is required for the virulence of *F. oxysporum* f.sp. cubense tropical race 4 toward the Cavendish banana (An et al., 2019). A mutation in *FocCP1* dramatically decreased the aerial growth and virulence of *F. oxysporum* f. sp. Cubense Tropical Race 4 (Liu et al., 2019). Fusaric acid plays a key role in the virulence of *F. oxysporum* toward host plants (López-Díaz et al., 2018). Meanwhile, ZafA-mediated regulation of zinc homeostasis is required for the virulence of *F. oxysporum* (López-Berges, 2020). These findings indicate the complex regulatory mechanism of *F. oxysporum* of controlling its growth and infection of different host plants. However, how *F. oxysporum* regulates its virulence toward tobacco is unknown.

Comparative transcriptome studies have been extensively performed in understanding host defense mechanism; however, they have rarely been used to dissect the virulence mechanisms of plant pathogens. Comparative transcriptomic analyses of four different infection stages of *Zymoseptoria tritici* identified significantly expressed genes during its infection of host plants (Palma-Guerrero et al., 2017), suggesting that this is a promising method to characterize virulence mechanisms of plant pathogens. In this study, we isolated 200 *F. oxysporum* strains from Yunnan Province, one of the main tobacco cultivating areas in China. Subsequently, the strains were classified into three groups according to their virulence level toward tobacco plants. In addition, comparative transcriptome analysis was performed to identify virulence associated genes, and *ATP synthase* genes were further analyzed their function in *F. oxysporum* infection to tobacco. These analyses provide important information opening up new avenues to study the mechanism of virulence of *F. oxysporum* during its interaction with tobacco.

## Materials and methods

### Isolation of *Fusarium oxysporum* strains

Fungal pathogens were isolated from tobacco plants with root rot disease in Kunming city, Yunnan Province of China. The pathogens were grown on the potato dextrose agar (PDA) plants and single spore collected for classify each strain. The DNA was extracted from each strain hypha, and the pathogens were further characterize by PCR using the Elongation factor (EF) gene (Geiser et al., 2004) specific primers (EF-1H: 5′-ATGGGTAAGGAAGACAAGAC-3′, EF-2T: 5′-GGAAGTACCAGTGATCATGTT-3′) to identify the *F. oxysporum* strains after sequencing of the PCR products. The PCR product sequences were matched in the NCBI^1^ and the Fusarum database.^2^ Sequences with greater than 99% identity with *F. oxysporum* sequences in the databases were deemed to be *F. oxysporum* strains.

### Pathogen cultivation and inoculation

The *F. oxysporum* strains were cultured on potato dextrose agar (PDA) medium in petri dishes. After 4 days of the *F. oxysporum* growth on the PDA plates, the hypha was collected and RNA was extracted. For the inoculation of tobacco plants, the *F. oxysporum* were cultured in liquid potato dextrose medium to generate spores, followed by filtration using 3M paper. The 1 ml of 1 × 10^–6^ of spores was injected into the soil 1 cm lower part from root base. following infection, the root and shoot apices were collected for RNA extraction. The tobacco plants were photographed and evaluated disease class after 7 days of inoculation.

### RNA extraction and RealTime Quantitative PCR

Total RNA was isolated using the RNeasy Plant Mini Kit (QIAGEN, Duesseldorf, Germany) and treated with RQ-RNase free DNase (Promega, Madison, WI, United States) to remove contaminating genomic DNA. For cDNA synthesis, reverse transcriptase minus RNase H (Toyobo)^3^ was used, according to the manufacturer’s instructions. The products obtained in RealTime Quantitative PCR (RT-qPCR) were quantified using Illumina Research Quantity software, Illumina Eco 3.0 (Illumina, San Diego, CA, United States) and the values were normalized against the tubulin levels in the same samples. The primers used for qRT-PCR are listed in Supplementary Table 1.

### ATP activity measurement

*Fusarium oxysporum* strains were cultured on PDA medium, collected, and frozen in liquid nitrogen, followed by grinding of the hypha. ATP synthase activity was measured spectrophotometrically (Thermo Fisher Scientific) at 340 nm by coupling the production of ADP to the oxidation of NADH by pyruvate kinase and lactate dehydrogenase as described (Cong et al., 2014).

### RNA-sequencing analysis

For the RNA-seq analysis, *F. oxysporum* stains were collected that displayed either strong, moderate, or weak virulence. Three biological replicates from each strain were performed. Quality control, mapping, expression analysis, differential expression analysis, gene ontology (GO) and Kyoto Encyclopedia of Genes and Genomes (KEGG) enrichment analysis were processed with GENEWIZ (Suzhou, China). The detail method for bioinformatic analyses were followed the published report (Yuan et al., 2020). The sequencing raw data was deposited in the NCBI database, and accession No. is PRJNA859524.

### ATP synthase gene knock-out vector construction and fungal transformation

A vector for the replacement of the ATP synthase gene (*pDATPS*) was constructed following a previously published method (Madrid et al., 2003). The construct harbored a hygromycin B resistance (Hyg*^r^*) cassette interrupting the ATP synthase gene open reading frame (ORF). This bipartite gene-targeting substrate was used for transformation of *F. oxysporum* wild-type protoplasts, according to a protocol described previously (Di Pietro and Roncero, 1998). Hyg*^r^* transformants were routinely subjected to two consecutive rounds of single sporing and stored as microconidia at −80°C. The knock-out mutant was confirmed by RT-qPCR using ATP synthase gene specific primers.

### Statistical analyses

Statistical analyses were conducted using Prism 5.0 software (GraphPad, San Diego, CA, United States) with one-way analysis of variance (ANOVA) for comparison of significant differences between multiple groups. Differences between the groups were considered significant if *P* < 0.05.

## Results

### Isolation of *Fusarium oxysporum* strains from tobacco plants with root rot disease

**FIGURE 1:**
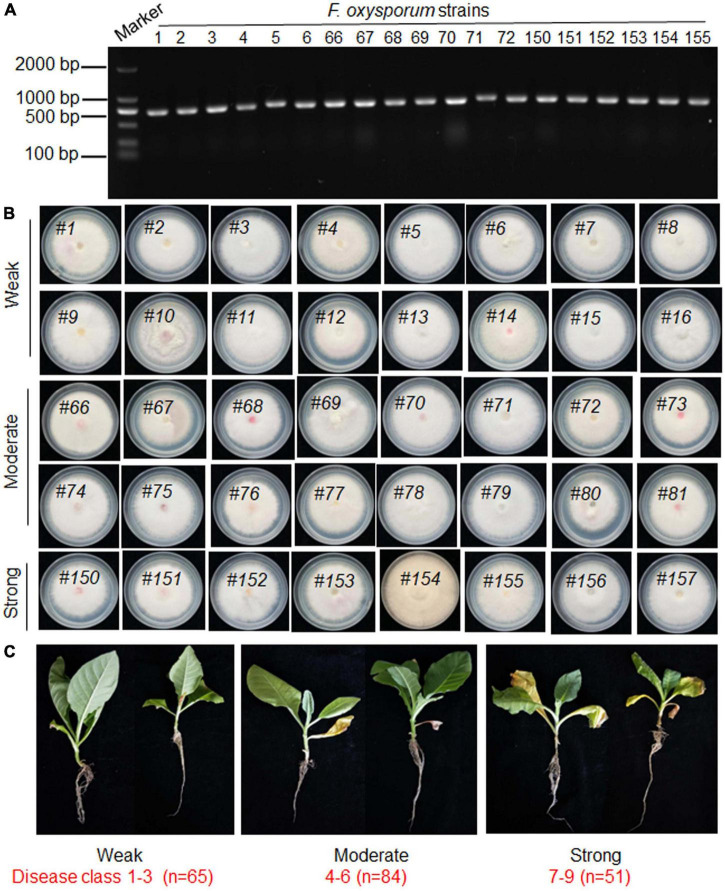
Isolation, identification, and virulence classification of *Fusarium oxysporum* strains. **(A)** The *Fusarium oxysporum* strains were identified by PCR using Elongation factor (EF) gene-specific primers EF-1H and EF-2T. A molecular size marker is shown at the left-hand side of the panel. The line numbers of each virulence type were labeled. **(B)** Representative images of *Fusarium oxysporum* isolates grown on PDA medium. The line numbers of each virulence type were labeled. **(C)** Representative images of tobacco plants inoculated with *Fusarium oxysporum* strains (total *n* = 200) displaying weak (disease class 1–3; *n* = 65), moderate (disease class 4–6; *n* = 84), and strong (disease class 7–9; *n* = 51) virulence.

To analyze the virulence mechanism of *F. oxysporum*, fungal samples were collected in a tobacco field in Kunming, Yunnan, that was affected by tobacco root rot. The isolated fungal pathogens were analyzed for the presence of *F. oxysporum* by PCR using the EF gene primers and subsequent sequencing (Figure 1A and Supplementary Table 2). In total, 200 strains of *F. oxysporum* were identified among 500 collected samples (Figure 1B). To classify their virulence strength toward tobacco plants, each strain was used to inoculate *Nicotiana tabacum* plants and the disease class was calculated. The 200 strains were classified into three groups based on their disease class. The strains from disease class 1–3 (*n* = 65) displayed weak virulence, class 4–6 (*n* = 84) displayed moderate virulence, while class 7–9 (*n* = 51) displayed strong virulence toward tobacco plants (Figure 1C).

### Comparative transcriptome analysis of *Fusarium oxysporum* stains with different virulence

**FIGURE 2:**
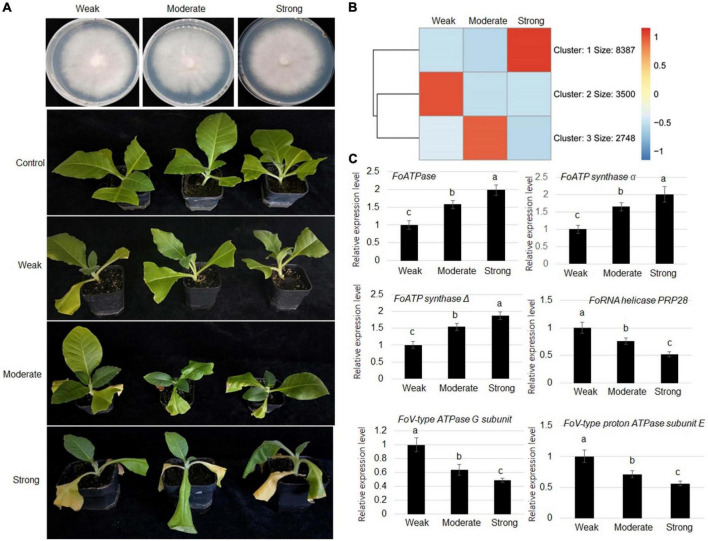
Comparative transcriptome analysis of *Fusarium oxysporum* strains displaying three levels of virulence (weak, moderate, and strong). **(A)** Representative images of three types of virulence *Fusarium oxysporum* strains grown on PDA medium. **(B)** Representative images of tobacco plants inoculated with *Fusarium oxysporum* strains displaying weak, moderate, or strong virulence, as indicated, toward tobacco plants. **(C)** Heat map of genes that are differentially expressed in *Fusarium oxysporum* strains displaying three levels of virulence. **(D)** Comparison of the expression levels of *FoATPase*, *FoATP synthase*α, *FoATP synthase*Δ, *FoRNA helicase PRP28*, *FoV-type ATPase G subunit*, and *FoV-type proton ATPase subunit E* in *Fusarium oxysporum* strains displaying different levels of virulence. Different letters above the bars indicate significant differences (*P* < 0.05).

To understand the virulence mechanism of *F. oxysporum* toward tobacco, strains with weak (class 1), moderate (class 5), and strong (class 9) virulence were collected for comparative transcriptome analysis (Figure 2A). The RNA-seq results showed that expression levels of 1,678 genes were positively correlated with virulence, while expression levels of 3,558 genes were negatively associated with the virulence (Figure 2B and Supplementary Tables 3, 4). To verify the RNA-seq analysis results, RT-qPCR was performed to verify gene expressions. The expression *FoATPase*, *FoATP synthaseα*, and *FoATP synthase*Δ genes was positively correlated with virulence and showed highest expression in a strong virulence line, while their expression was the lowest in the weak stain. Meanwhile, *FoRNA helicase PRP28*, *FoV-type ATPase G subunit*, and *FoV-type proton ATPase subunit E* gene expressions were negatively correlated with virulence (Figure 2C).

**FIGURE 3:**
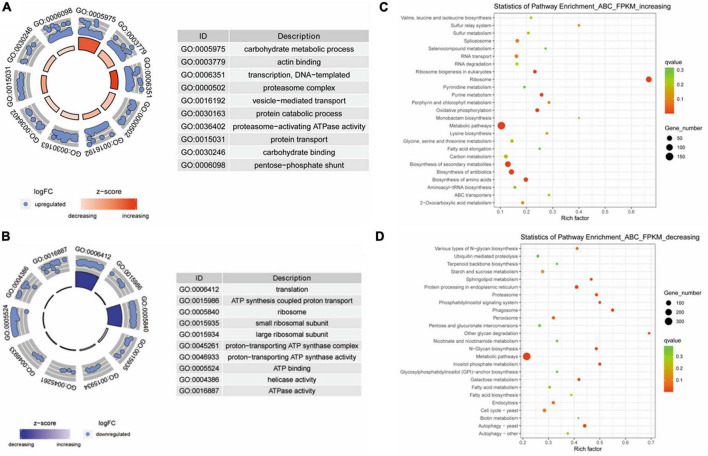
Gene ontology (GO) and Kyoto Encyclopedia of Genes and Genomes (KEGG) analyses of differentially expressed genes in *Fusarium oxysporum* strains with different levels of virulence toward tobacco. **(A)** GO analysis for genes that were negatively associated with virulence. These genes are enriched in the terms including carbohydrate metabolism, protein catabolic process, and carbohydrate binding. **(B)** Genes that were positively correlated with virulence show enrichment for the terms including ATP synthesis coupled proton transport, ATP binding, and ATPase activity. **(C)** KEGG analysis of the genes with negative correlation with virulence shows enrichment in categories including RNA transport, ribosome, and ABC transporters. **(D)** The genes with expression levels that positively correlated with virulence are classified into categories including proteasome, metabolic pathways, and autophagy.

Next, the differentially expressed genes were analyzed according to biological processes using GO and KEGG analyses. GO analysis showed that the expression levels of genes that negatively correlated with virulence are classified into processes that included carbohydrate metabolism, protein transport, and actin binding (Figure 3A). The expression of genes that positively correlated with virulence was enriched into the terms including ATP synthesis-coupled proton transport, ATP binding, and ATPase activity (Figure 3B). Furthermore, KEGG analysis showed that the expression of genes that were negatively correlated with virulence were enriched in categories that included RNA transport, ribosome, and ABC transporters (Figure 3C). The gene expression level with positive correlation with virulence was classified into categories including proteasome. Metabolic pathways, and autophagy (Figure 3D).

### ATP synthase gene expression and energy charge in *Fusarium oxysporum* stains with different virulence

**FIGURE 4:**
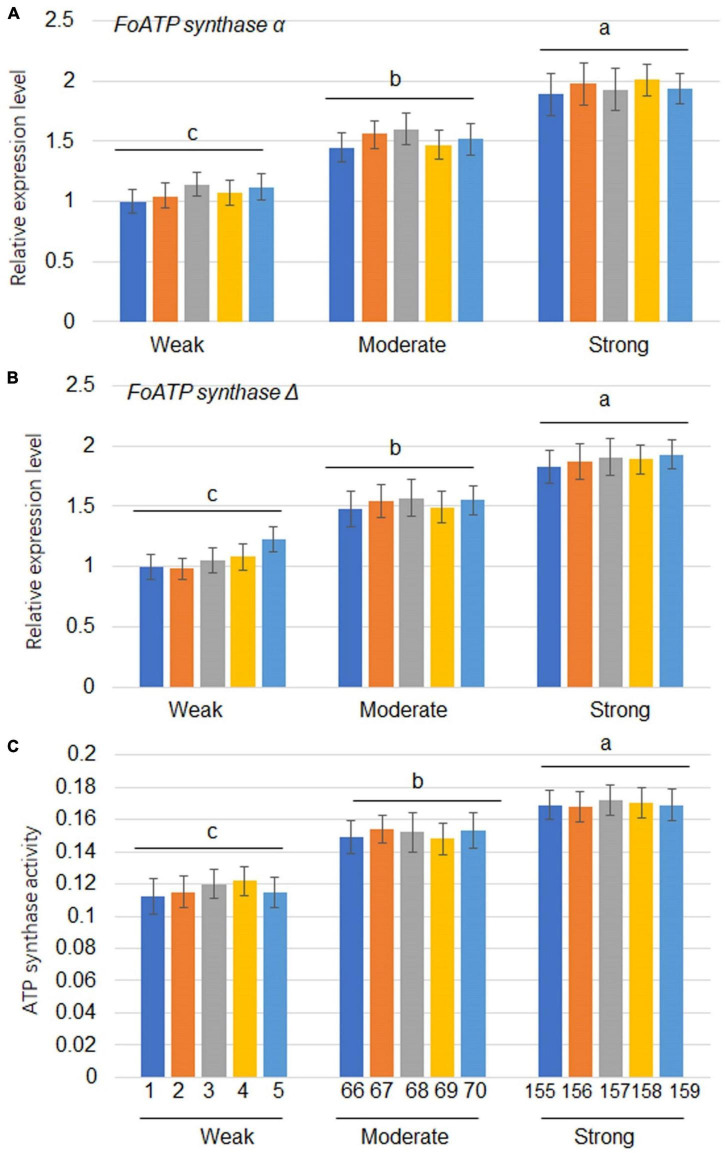
The expression levels of *ATP synthase*α and *ATP synthase*Δ as well as ATP synthase activity in *Fusarium oxysporum* strains with different levels of virulence. The expression levels of *FoATP synthase*α **(A)** and *FoATP synthase*Δ **(B)** in *F. oxysporum* strains displaying three different levels of virulence (weak, moderate, or strong; 5 strains for each type of virulence). **(C)** ATP synthase activity was measured in *F. oxysporum* strains displaying three different levels of virulence. The line numbers of each virulence type were the same as shown in Figure 1. Different letters above the bars indicate significant differences (*P* < 0.05).

Since the expression of ATP synthase genes was positively correlated with the levels of virulence of the *F. oxysporum* strains, 5 strains from each virulence group were tested for evaluation of ATP synthase gene expressions. The results showed that *FoATP synthaseα* and Δ gene expressions were positively correlated with virulence levels of the strains tested (Figures 4A,B). Expression levels of *FoATP synthase* α and Δ genes after weak, moderate, and strong virulence strains inoculation were examined. The results showed that *FoATP synthase* α and Δ gene expressions were induced after inoculation of three strains with the highest induction kinetic in the strong virulence strain inoculation group (Supplementary Figure 1). Next, the ATP synthase activity was calculated in the *F. oxysporum* stains. The results indicated that, similarly to the *ATP synthase* gene expression patterns, the ATP synthase activity was positively correlated with the virulence levels of *F. oxysporum* (Figure 4C).

### *ATP synthase* α positively regulates *Fusarium oxysporum* virulence to tobacco plants

**FIGURE 5:**
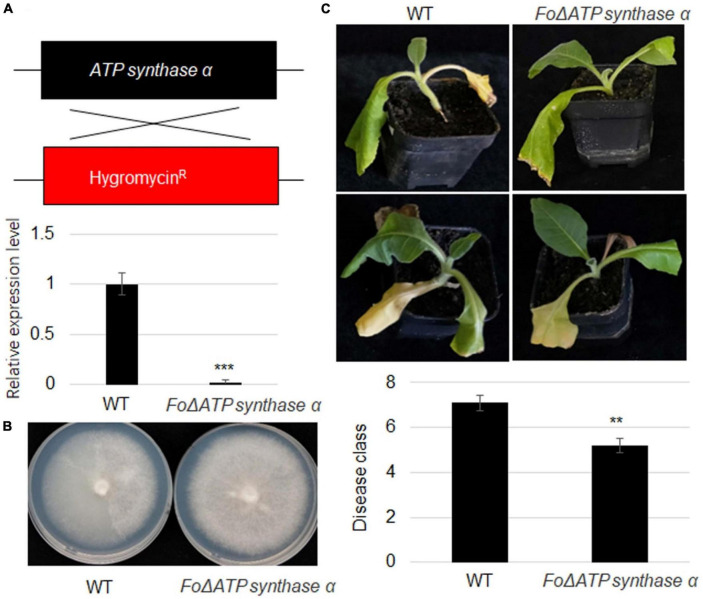
An *ATP synthase*α mutation in *Fusarium oxysporum* severely reduces its virulence toward tobacco plants. **(A)** Scheme of recombination of *FoATP synthase* and hygromycin fragment in Δ*ATP synthase*α mutant and relative expression level of *FoATP synthase*. **(B)** Representative images of wild-type and Δ*FoATP synthase* mutant *Fusarium oxysporum* strains grown on PDA medium. **(C)** The phenotype of tobacco plants upon inoculation of WT and Δ*FoATP synthase* mutant strains of *Fusarium oxysporum*. The disease class of both types of tobacco plants was calculated, **P < 0.01; ***P < 0.001.

To further investigate *ATP synthase*α function, its mutant strain was generated using homologs recombination approach in a strong virulence strain. The *pDATPS* was transformed into *F. oxysporum* protoplast, and hygromycin resistant (Hyg*^r^*) transformants were collected. RT-qPCR result showed that *ATP synthase* α was not detected in the mutant strain (Figure 5A). The growth of Δ*FoATP synthase* α was similar with wild-type strain (Figure 5B). However, inoculation of wild-type and mutant strain identified that *FoATP synthase* α knock-out reduced virulence of *F. oxysporum* to tobacco plants (Figure 5C).

**FIGURE 6:**
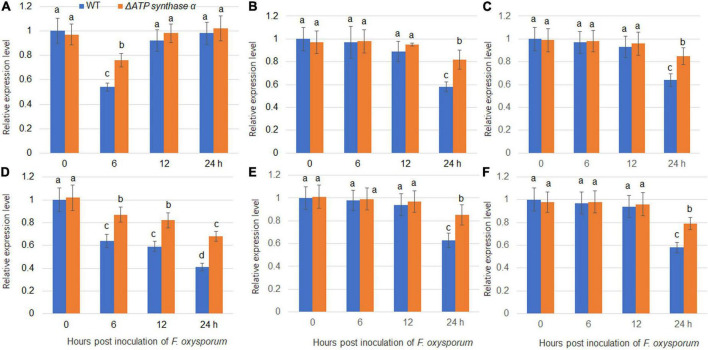
Expression of sugar transporters in tobacco plants following inoculation with wild-type and Δ*FoATP synthase*α mutant strains of *Fusarium oxysporum*. The expression of *NtSUC4* **(A)**, *NtSTP12* **(B)**, *NtHEX6* **(C)**, *NtSWEET1* **(D)**, *NtSWEET3b* **(E)**, and *NtSWEET12* **(F)** in tobacco plants was examined 0-, 6-, 12-, and 24-h post inoculation with wild-type (blue bars) and Δ*ATP synthase*α mutant (red bars) strains. Different letters above the bars indicate significant differences (*P* < 0.05).

Our previous study found that *F. oxysporum* inoculation suppressed expression levels of several sugar transporters including *NtSUC4*, *NtSTP12*, *NtHEX6*, *NtSWEET1*, *NtSWEET3b*, and *NtSWEET12* (Gai et al., 2021). We therefore analyzed the gene expression levels of sugar transporters in wild-type and Δ*FoATP synthase* α inoculated tobacco plants. The results showed that *NtSUC4*, *NtSTP12*, *NtHEX6*, *NtSWEET1*, *NtSWEET3b*, and *NtSWEET12* expression levels were suppressed by inoculation of both wild-type and Δ*ATP synthase* α, but that their expression levels were higher in the Δ*FoATP synthase* α group compared to the wild-type inoculated group (Figure 6). In addition, *NtSUC4*, *NtSTP12*, *NtHEX6*, *NtSWEET1*, *NtSWEET3b*, and *NtSWEET12* expression levels were monitored after inoculation of the weak, moderate, and strong virulence lines. The results showed these sugar transporters were suppressed by inoculation of three types of virulence strains with different kinetics, and the highest suppression kinetic was shown in strong virulence strain inoculation group (Supplementary Figure 2).

## Discussion

Tobacco root rot is caused by infection of Fusarium isolates, which severely affects tobacco growth. Fusarium is a soil-born fungal pathogen that is hard to control; therefore, fungicides are the main source to protect plants from *Fusarium*. *F. oxysporum* is a typical soilborne fungus which colonize plant vasculature to result in serious crop wilt disease (de Sain and Rep, 2015). *F. oxysporum* might propagate in the plant vasculature to block water or nutrient translocation or secrete some enzymes to hydrolyze plant cell wall. The potential mechanism against of virulence of Fusarium has been analyzed. Mycotoxins are the main virulence molecules from *F. graminearum*, and their biosynthesis and regulation has been well studied (Chen et al., 2019). In addition, secreted proteins were identified that modulate host plant defense and *F. oxysporum* virulence (Chen et al., 2014; Liu et al., 2019; Ding et al., 2020; Gurdaswani et al., 2020). However, the molecular mechanism of *F. oxysporum* virulence toward tobacco plants remains unclear.

To investigate the mechanism of *F. oxysporum* virulence toward tobacco plants, fungal pathogen samples were collected from tobacco plants with root rot disease in Kunming, Yunnan province, China. The 500 samples that were collected were analyzed for the presence of *F. oxysporum* by PCR using EF gene specific primers and a subsequent sequencing approach. The data suggest that a high proportion (40%) of the collected samples contained *F. oxysporum* (data not shown), implying that the main causative agent of tobacco root rot in that region is *F. oxysporum*. Furthermore, the 200 *F. oxysporum* strains were used to inoculate *N. tabacum* plants to allow their classification according to virulence levels. Based on the disease levels observed on the tobacco seedlings, the 200 strains were grouped into three classes, showing either weak (65), moderate (81), or strong (51) virulence toward tobacco plants. Next, transcriptome analysis was carried out comparing *F. oxysporum* strains with different levels of virulence toward tobacco. The RNA-seq results identified 1,678 and 3,558 genes that were positively and negatively associated with the virulence levels, respectively, suggesting that a large number of genes are involved in the virulence phenotypes. Furthermore, GO and KEGG classification indicated that the differentially expressed genes are involved in diverse biological process, highlighting the complicated regulatory mode of *F. oxysporum* virulence. The GO analysis showed that greater number of genes classified into terms including ATP binding, ATP synthesis coupled proton transport, and ATPase activity. Also, interestingly, the expression levels of *FoATP synthase* alpha and delta genes were positively correlated with *F. oxysporum* virulence. Additionally, ATP synthase gene expression and energy charge values were calculated from 5 strains each belonging to the weak, moderate, and strong virulence group to evaluate whether this is common mechanism by which the *F. oxysporum* virulence is modulated. Also, *FoATP synthase* genes were induced upon weak, moderate, and strong virulence stain inoculation with the stronger induction ratio in the strong virulence strain inoculation group than in weak and moderate virulence strain inoculation groups. The data suggested that *FoATP synthase* expression levels and energy charge are positively correlated with virulence types. Inoculation of tobacco plants with wild-type and *FoATP synthase alpha* gene mutated *F. oxysporum* further revealed that ATP content is important for *F. oxysporum* virulence. ATP is the energy source for organism which support normal action of multiple biological processes. Also, extracellular ATP was also identified as the signal molecule in plants (Choi et al., 2014), and ATP signaling plays an important role in plant response to environmental changes including stress response (Cao et al., 2014). Extracellular ATP trigger cellular calcium response to activate calcium-dependent signaling transduction. Also, ATP signal via its receptor DORN1 (Does not Respond to Nucleotides 1) to activate NADPH oxidase gene *RBOH* expressions as well as activate MPK3 and MPK6 in *Arabidopsis* (Choi et al., 2014). These findings suggest that extracellular ATP-mediated signaling is important for plant response to environmental changes and plant growth and development. Therefore, further analyses are required to clarify the molecular mechanism of how ATP content and signaling modulate *F. oxysporum* virulence toward tobacco.

Previously, we reported that *F. oxysporum* infection suppressed sugar transporters including SUC, STP, HEX, and SWEET family members in tobacco (Gai et al., 2021). Also, six sugar transporter genes were suppressed with different kinetics with stronger suppression ratio in the strong virulence inoculation group than in weak and moderate virulence stains inoculation groups. To further evaluate the role of ATP synthesis in the regulation of *F. oxysporum* virulence, tobacco plants were inoculated with wild-type and *FoATP synthase alpha* mutated strains. As expected, the sugar transporter expression levels were insensitive to *FoATP synthase alpha* mutated compared to wild-type *F. oxysporum*, suggesting the suppression of sugar transporter expression was associated with virulence. Sugar transporters have been reported to play key roles during host-pathogen interaction (Tadege et al., 1998; Asai et al., 2016; Devanna et al., 2021), and the suppression of sugar transporters might regulate the distribution of sugar, causing the starvation of the *F. oxysporum*.

This study used comparative transcriptome analysis to investigate the virulence mechanism of *F. oxysporum*. The results suggest a complex regulatory mode of control of *F. oxysporum* virulence, and ATP synthesis might be an important pathway for partially modulating the virulence. The information presented here is important for a further understanding of the molecular basis of *F. oxysporum* virulence toward tobacco plants.

## Data availability statement

The original contributions presented in this study are included in the article/Supplementary material, further inquiries can be directed to the corresponding authors.

## Author contributions

XG, YX, and ZX conceived and designed the sequence data analysis and wrote the manuscript. SL performed the bioinformatic analysis. XG, NJ, and QS contributed to data analysis. All authors read and approved the final manuscript.

## Footnotes

1
http://blast.ncbi.nlm.nih.gov/Blast.cgi

2
http://isolate.fusariumdb.org

3
http://www.toyobo-global.com/
