# Supplementary material for: Comparative transcriptome analysis reveals that ATP synthases regulate Fusarium oxysporum virulence by modulating sugar transporter gene expressions in tobacco
Source: Front Plant Sci. 2022 Aug 18;13:978951. doi: 10.3389/fpls.2022.978951 (PMC9433920; doi:10.3389/fpls.2022.978951)
Supplement: Supplementary file 1 [file Data_Sheet_1.DOCX]

**Supplemental Figures**

**
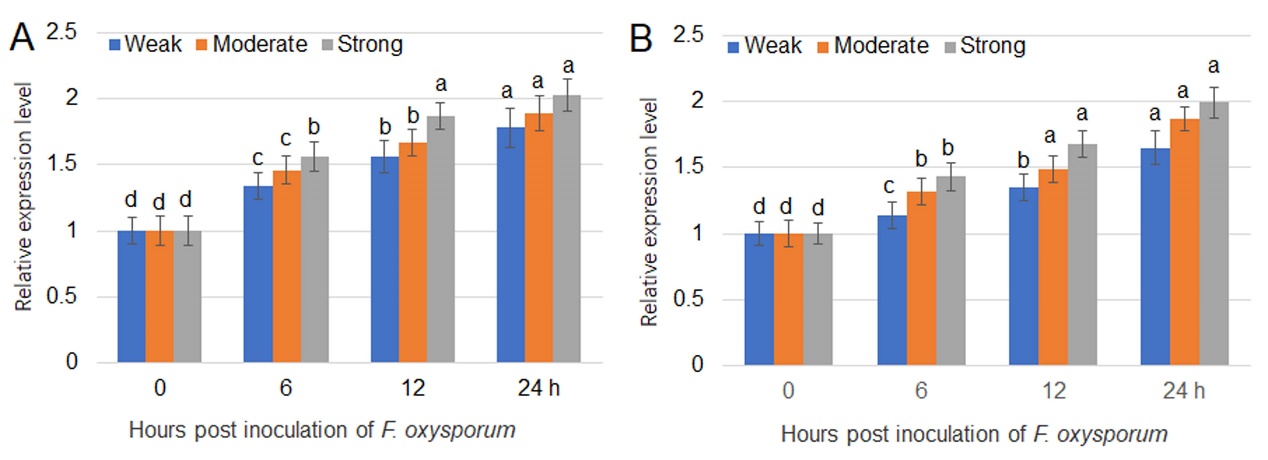
**

**FIGURE S1|** The expression levels of *ATP synthase α* and *ATP synthase Δ* genes after inoculation of three virulence type strains. (A) *ATP synthase α* and (B) *ATP synthase Δ* expression levels after 0, 6, 12, and 24 hours of weak, moderate, and strong virulence stains inoculation. Different letters above the bars indicate significant differences (*P<0.05*).

**
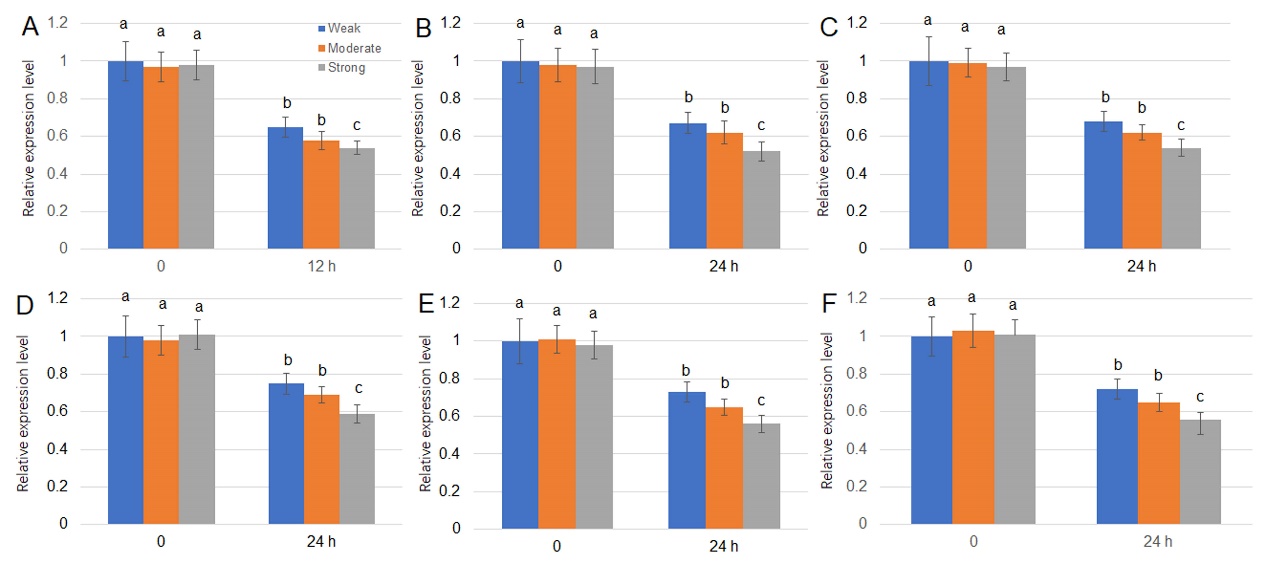
**

**FIGURE S2|** Expression of sugar transporters in tobacco plants following inoculation with the weak, moderate, and strong virulence strains of *F. oxysporum*. The expression of *NtSUC4* (A), *NtSTP12* (B), *NtHEX6* (C), *NtSWEET1* (D), *NtSWEET3b* (E), and *NtSWEET12* (F) in tobacco plants was examined 0-, 12-, or 24-hours post inoculation with the weak (blue bars), moderate (orange bars), and strong virulence (gray bars) strains. Different letters above the bars indicate significant differences (*P* < 0.05).
