# Supplementary material for: Comparative transcriptome analysis reveals that ATP synthases regulate Fusarium oxysporum virulence by modulating sugar transporter gene expressions in tobacco
Source: Front Plant Sci. 2022 Aug 18;13:978951. doi: 10.3389/fpls.2022.978951 (PMC9433920; doi:10.3389/fpls.2022.978951)
Supplement: Supplementary file 2 [file Table_1.DOCX]

Supplemental Table S2. The partial sequencing results of *EF* genes amplified by PCR from Fusarum isolates

>21LC5 Fusarium oxysporum 99.86%

GGAAGTACCAGTGATCATGTTCTTGATGAAATCACGGTGACCGGGAGCGTCTGAGTGATATGTTAGTACGAAGAGAAGTAGAATGAAGCATGAGCGACAACATACCAATGACGGTGACATAGTAGCGAGGAGTCTCGAACTTCCAGAGAGCAATATCGATGGTGATACCACGCTCACGCTCGGCCTTGAGCTTGTCAAGAACCCAGGCGTACTTGAAGGAACCCTTACCGAGCTCAGCGGCTTCCTATTGTTGAATGGTTAGTGACTGCTTGACACGTGACGACGCACTCATTGAGGTTCTGAGAATGGTAAGAGGGCAAACGCTCCCGTCGCTCAAGAGGCGGGGTAAGTGCCCCACCAAAAAAATTACGGTCATATTGCAAAATTTTTGGTCTCGAGCGGGGTAGCGGGCACGTTTCGAGTCGTAGGGGAAATCGATGGGCAAAGGACGCGCGATTGAAGGGAAAGTGACTAACCTTCTCGAACTTCTCGATGGTTCGCTTGTCGATACCACCGCACTGGTAGATCAAGTGACCGGTCTGTGAAACGATGTCAGTATGTTGACTTTGAGAAATACCCCGCCAGGTCTTGGTCGGGATTGACGATGGCAGATATGCTCATTGTCGAGGAGAGTACTCACAGTGGTCGACTTGCCAGAGTCGACGTGGCCGATGACGACGACGTTAAGGTGAGTCTTGTCTTCCTTACCCAT

>21TH19 Fusarium oxysporum 99.72%

GGAAGTACCAGTGATCATGTTCTTGATGAAATCACGGTGACCGGGAGCGTCTGAGTGATGTTAGTACGAAGAGAAGTAGAATGAAGCATGAGCGACAACATACCAATGACGGTGACATAGTAGCGAGGAGTCTCGAACTTCCAGAGAGCAATATCGATGGTGATACCACGCTCACGCTCGGCCTTGAGCTTGTCAAGAACCCAGGCGTACTTGAAGGAACCCTTACCGAGCTCAGCGGCTTCCTATTGTTGAATGGTTAGTGACTGCATGACACGTGACGACGCACTCATTGAGGTTGTGAGAATGGTTAAGAGGGCAAACGCTCCCGTCGCTCAAGTGGCGGGGTAAGTGCCCCACCAAAAAAATTACAGTCATATTGCAAAATTTTTGGTCTCGAGCGGGGTAGCGGGCACGTTTCGAGTCGTAGGGGAAATCGATGGGCAAAGGACGCGCGATCGAAGGGAAAGTGACTAACCTTCTCGAACTTCTCGATGGTTCGCTTATCGATACCACCGCACTGGTAGATCAAGTGACCGGTCTGTGAAACGATGTCAGTATGTTGACTTTGAGAAATACCCCACCAGGTCTTGGTCGGGATTGACGATGGCAGATAAGCTCATTGTCGAGGAGAGTACTCACAGTGGTCGACTTGCCAGAGTCGACGTGGCCGATGACGACGACGTTAAGGTGAGTCTTGTCTTCCTTACCCAT

>21TH21 Fusarium oxysporum 99.86%

GGAAGTACCAGTGATCATGTTCTTGATGAAATCACGGTGACCGGGAGCGTCTGAGTGATATATTAGTACGAAGAGAAGTAGAATGAAGCATGAGCGACAACATACCAATGACGGTGACATAGTAGCGAGGAGTCTCGAACTTCCAGAGAGCAATATCGATGGTGATACCACGCTCACGCTCGGCCTTGAGCTTGTCAAGAACCCAGGCGTACTTGAAGGAACCCTTACCGAGCTCAGCGGCTTCCTATTGTTGAATGGTTAGTGACTGCTTGACACGTGACGACGCACTCATTGAGGTTGTGATGGTAAGAGGGCAAACGCGCCCCGTCGCTCAAGTGGCGTAGTAAATGCCCCACCAAAAAAATTACGGTCATATTGCAAAATTTTTGGTCTCGAGCGGGGTAGCGGGCACGTTTCGAGTCGTAGGGGAAGTCGATGGGCAAAGGACGCGCGATCGAAGGGAAAGTGACTAACCTTCTCGAACTTCTCGATGGTTCGCTTGTCGATACCACCGCACTGGTAGATCAAGTGACCGGTCTGTGAAACGATGTCAGTATGTTGCCTTTGAGAAGTACCCCGCCAGGTCTTGGTCGGGATTGACGATGGCAGATAAGCTCATTGTCGAGGAGAGTACTCACAGTGGTCGACTTGCCAGAGTCGACGTGGCCGATGACGACGACGTTAAGGTGAGTCTTGTCTTCCTTACCCAT

>21YH22 Fusarium oxysporum 100%

GGAAGTACCAGTGATCATGTTCTTGATGAAATCACGGTGACCGGGAGCGTCTGAGTGATATGTTAGTACGAAGAGAAGTAGAATGAAGCATGAGCGACAACATACCAATGACGGTGACATAGTAGCGAGGAGTCTCGAACTTCCAGAGAGCAATATCGATGGTGATACCACGCTCACGCTCGGCCTTGAGCTTGTCAAGAACCCAGGCGTACTTGAAGGAACCCTTACCGAGCTCAGCGGCTTCCTATTGTTGAATGGTTAGTGACTGCTTGACACGTGACGACGCACTCATTGAGGTTCTGAGAATGGTAAGAGGGCAAACGCTCCCGTCGCTCAAGTGGCGGGGTAAGTGCCCCACCAAAAAAATTACGGTCATATTGCAAAATTTTTGGTCTCGAGCGGGGTAGCGGGCACGTTTCGAGTCGTAGGGGAAATCGATGGGCAAAGGACGCGCGATTGAAGGGAAAGTGACTAACCTTCTCGAACTTCTCGATGGTTCGCTTGTCGATACCACCGCACTGGTAGATCAAGTGACCGGTCTGTGAAACGATGTCAGTATGTTGACTTTGAGAAATACCCCGCCAGGTCTTGGTCGGGATTGACGATGGCAGATATGCTCATTGTCGAGGAGAGTACTCACAGTGGTCGACTTGCCAGAGTCGACGTGGCCGATGACGACGACGTTAAGGTGAGTCTTGTCTTCCTTACCCAT

>21XP25 Fusarium oxysporum 99.86%

GGAAGTACCAGTGATCATGTTCTTGATGAAATCACGGTGACCGGGAGCGTCTGGGTGATATGTTAGTACGAAGAGAAGTAGAATGAAGCATGAGCGACAACATACCAATGACGGTGACATAGTAGCGAGGAGTCTCGAACTTCCAGAGAGCAATATCGATGGTGATACCACGCTCACGCTCGGCCTTGAGCTTGTCAAGAACCCAGGCGTACTTGAAGGAACCCTTACCGAGCTCAGCGGCTTCCTATTGTTGAGTGGTTAGTGACTGCTTGACACGTGACGACGCACTCATTGAGGTTGTGAGAATGGTAAGAGGGCAAACGCTCCCGTCGCTCAAGTGGCGGGGTAAGTGCCCCACCAAAAAAATTACGGTCATATTGCAAAATTTTTGGTCTCGAGCGGGGTAGCGGGCACGTTTCGAGTCGTAGGGGAAATCGATGGGCAAAGGACGCGCGATTGAAGGGAAAGTGACTAACCTTCTCGAACTTCTCGATGGTTCGCTTGTCGATACCACCGCACTGGTAGATCAAGTGACCGGTCTGTGAAACGATGTCAGTATGTTGACTTTGAGAAATACCCCGCCAGGTCTTGGTCGGGATTGACGATGGCAGATATGCTCATTGTCGAGGAGAGTACTCACAGTGGTCGACTTGCCAGAGTCGACGTGGCCGATGACGACGACGTTAAGGTGAGTCTTGTCTTCCTTACCCAT

>21LC27 Fusarium oxysporum 100%

GGAAGTACCAGTGATCATGTTCTTGATGAAATCACGGTGACCGGGAGCGTCTGAGTGATATATTAGTACGAAGAGAAGTAGAATGAAGCATGAGCGACAACATACCAATGACGGTGACATAGTAGCGAGGAGTCTCGAACTTCCAGAGAGCAATATCGATGGTGATACCACGCTCACGCTCGGCCTTGAGCTTGTCAAGAACCCAGGCGTACTTGAAGGAACCCTTACCGAGCTCAGCGGCTTCCTATTGTTGAATGGTTAGTGACTGCTTGACACGTGACGACGCACTCATTGAGGTTGTAATGGTAAGAGGGCAAACGCGCCCCGTCGCTCAAGTGGCGGGGTAAATGCCCCACCAAAAAAATTACGATCATATTGCAAAATTTTTGGTCTCGAGCGGGGTAGCGGGCACGTTTCGAGTCGTAGGGGAAGTCGATGGGCAAAGGACGCGCGATCGAAGGGAAAGTGACTAACCTTCTCGAACTTCTCGATGGTTCGCTTGTCGATACCACCGCACTGGTAGATCAAGTGACCGGTCTGTGAAACGATGTCAGTATGTTGCCTTTGAGAAGTACCCCGCCAGGTCTTGGTCGGGATTGACGATGGCAGATAAGCTCATTGTCGAGGAGAGTACTCACAGTGGTCGACTTGCCAGAGTCGACGTGGCCGATGACGACGACGTTAAGGTGAGTCTTGTCTTCCTTACCCAT

>21DL35 Fusarium oxysporum 99.86%

GGAAGTACCAGTGATCATGTTCTTGATGAAATCACGGTGACCGGGAGCGTCTGAGTGATATGTTAGTACGAAGAGAAGTAGAATGAAGCATGAGCGACAACATACCAATGACGGTGACATAGTAGCGAGGAGTCTCGAACTTCCAGAGAGCAATATCGATGGTGATACCACGCTCACGCTCGGCCTTGAGCTTGTCAAGAACCCAGGCGTACTTGAAGGAACCCTTACCGAGCTCAGCGGCTTCCTATTGTTGAATGGTTAGTGACTGCTTGACACGTGACGACGCACTCATTGAGGTTGTGAGAATGGTAAGAGGGCAAACGCTCCCGTCGCTCAAGTGGCGGGGTAAGTGCCCCACCAAAAAAATTACGGTCATATTGCAAAAATTTTGGTCTCGAGCGGGGTAGCGGGCACGTTTCGAGTCGTAGGGGAAATCGATGGGCAAAGGACGCGCGATTGAAGGGAAAGTGACTAACCTTCTCGAACTTCTCGATGGTTCGCTTGTCGATACCACCGCACTGGTAGATCAAGTGACCGGTCTGTGAAACGATGTCAGTATGTTGACTTTGAGAAATACCCCGCCAGGTCTTGGTCGGGATTGACGATGGCAGATATGCTCATTGTCGAGGAGAGTACTCACAGTGGTCGACTTGCCAGAGTCGACGTGGCCGATGACGACGACGTTAAGGTGAGTCTTGTCTTCCTTACCCAT

>21DL44 Fusarium oxysporum 99.44%

GGAAGTACCAGTGATCATGTTCTTGATGAAATCACGGTGACCGGGAGCGTCTGAGTGATATGTTAGTACGAAGAGAAGTAGAATGAAGCATGAGCGACAACATACCAATGACGGTGACATAGTAGCGAGGAGTCTCGAACTTCCAGAGAGCAATATCGATGGTGATACCACGCTCACGCTCGGCCTTGAGCTTGTCAAGAACCCAGGCGTACTTGAAGGAACCCTTACCGAGCTCAGCGGCTTCCTATTGTTGAATGGTTAGTGACTGCTTGACACGTGACGACGCACTCATTGAGGTTGTGAGAATGGTAAGAGGGCAAACGCTCCCGTCGCTCAAGTGGCGGGGTAAGTGCCCCACCAAAAAAAATTACCGTCATATTGCAAAATTTTTGGTCTCGAGCGGGGTAGCGGGCACGTTTCGAGTCGTAGGGGAAATCGATGGGCAAAGGACGCGCGATCGAAGGGAAAGTGACTAACCTTCTCGAACTTCTCGATGGTTCGCTTGTCGATACCACCGCACTGGTAGATCAAGTGACCGGTCTGTGAAACGATGTCAGTATGTTGACTTTGACAAATACCCCGCCAGGTCTTGGTCGGGACTGACGATGGCAGATAAGCTCATTGTCGAGGAGAGTACTCACAGTGGTCGACTTGCCAGAGTCGACGTGGCCGATGACGACGACGTTAAGGTGAGTCTTGTCTTCCTTACCCAT

>21SL52 Fusarium oxysporum 99.86%

GGAAGTACCAGTGATCATGTTCTTGATGAAATCACGGTGACCGGGAGCGTCTGAGTGATATGTTAGTACGAAGAGAAGTAGAATGAAGCATGAACGACAACATACCAATGACGGTGACATAGTAGCGAGGAGTCTCGAACTTCCAGAGAGCAATATCGATGGTGATACCACGCTCACGCTCGGCCTTGAGCTTGTCAAGAACCCAGGCGTACTTGAAGGAACCCTTACCGAGCTCAGCGGCTTCCTATTGTTGAATGGTTAGTGACTGCTTGACACGTGACGACGCACTCATTGAGGTTGTGAGAATGGTAAGAGGGCAAACGCTCCCGTCGCTCAAGTGGCGGGGTAAGTGCCCCACCGAAAAAAATTACGGTCATATTGCAAAATTTTTGGTCTCGAGCGGGGTAGCGGGCACGTTTCGAGTCGTAGGGGAAATCGATGGGCAAAGGACGCGCGATCGAAGGGAAAGTGACTAACCTTCTCGAACTTCTCGATGGTTCGCTTGTCGATACCACCGCACTGGTAGATCAAGTGACCGGTCTGTGAAACGATGTCAGTATGTTGACTTTGAGAAATACCCCGCCAGGTCTTGGTCGGGATTGACGATGGCAGATAAGCTCATTGTCGAGGAGAGTACTCACAGTGGTCGACTTGCCAGAGTCGACGTGGCCGATGACGACGACGTTAAGGTGAGTCTTGTCTTCCTTACCCAT

>21ZT73 Fusarium oxysporum 99.72%

GGAAGTACCAGTGATCATGTTCTTGATGAAATCACGGTGACCGGGAGCGTCTGGGTGATATGTTAGTACGAAGAGAAGTAGAATGAAGCATGAGCGACAACATACCAATGACGGTGACATAGTAGCGAGGAGTCTCGAACTTCCAGAGAGCAATATCGATGGTGATACCACGCTCACGCTCGGCCTTGAGCTTGTCAAGAACCCAGGCGTACTTGGAGGAACCCTTACCGAGCTCAGCGGCTTCCTATTGTTGAGTGGTTAGTGACTGCTTGACACGTGACGACGCACTCATTGAGGTTGTGAGAATGGTAAGAGGGCAAACGCTCCCGTCGCTCAAGTGGCGGGGTAAGTGCCCCACCAAAAAAATTACGGTCATATTGCAAAATTTTTGGTCTCGAGCGGGGTAGCGGGCACGTTTCGAGTCGTAGGGGAAATCGATGGGCAAAGGACGCGCGATTGAAGGGAAAGTGACTAACCTTCTCGAACTTCTCGATGGTTCGCTTGTCGATACCACCGCACTGGTAGATCAAGTGACCGGTCTGTGAAACGATGTCAGTATGTTGACTTTGAGAAATACCCCGCCAGGTCTTGGTCGGGATTGACGATGGCAGATATGCTCATTGTCGAGGAGAGTACTCACAGTGGTCGACTTGCCAGAGTCGACGTGGCCGATGACGACGACGTTAAGGTGAGTCTTGTCTTCCTTACCCAT

>21SL93 Fusarium oxysporum 100%

GCCTTTCCCAAAAAAGGGGTTCCGGGGCCTTTGTTCCGGGGTTGGGAGGCGGGAAACCTTGACCATGCAGTTCCGGGACCGTCCAATTTTTTGTAACGGATCCGGAGCCGCCAAGCCGTCAGGCCCGTCACCGGGTTTGCCGGGTTCGGGCTTGCTTAATATGCCGCATCAGACCAGATTTTCTGAGAGTCCCCATATGCGGTTGAAATCCCGCACAGATGCGTAAGGAGAAAATACCGCATCAGGCGCCATTCGCCATTCAGGCTGCGCAACTGTGGGGAAGGGCGATCGGTGCGGGCCTCTTCGCTATTACGCCAGCTGGCGAAAGGGGGATGTGCTGCAAGGCGATTAAGTTGGGTAACGCCAGGGTTTTCCCAGTCACGACGTTGTAAAACGACGGCCAGTGAATTCGAGCTCGGTACCCGGGGATCCTCTAGAGATTATGGGTAAGGAAGACAAGACTCACCTTAACGTCGTCGTCATCGGCCACGTCGACTCTGGCAAGTCGACCACTGTGAGTACTCTCCTCGACAATGAGCATATCTGCCATCGTCAATCCCGACCAAGACCTGGCGGGGTATTTCTCAAAGTCAACATACTGACATCGTTTCACAGACCGGTCACTTGATCTACCAGTGCGGTGGTATCGACAAGCGAACCATCGAGAAGTTCGAGAAGGTTAGTCACTTTCCCTTCAATCGCGCGTCCTTTGCCCATCGATTTCCCCTACGACTCGAAACGTGCCCGCTACCCCGCTCGAGACCAAAAATTTTGCAATATGACCGTAATTTTTTTGGTGGGGCACTTACCCCGCCACTTGAGCGACGGGAGCGTTTGCCCTCTTACCATTCTCAGAACCTCAATGAGTGCGTCGTCACGTGTCAAGCAGTCACTAACCATTCAACAATAGGAAGCCGCTGAGCTCGGTAAGGGTTCCTTCAAGTACGCCTGGGTTCTTGACAAGCTCAAGGCCGAGCGTGAGCGTGGTATCACCATCGATATTGCTCTCTGGAAGTTCGAGACTCCTCGCTACTATGTCACCGTCATTGGTATGTTGTCGCTCATGCTTCATTCTACTTCTCTTCGTACTAACATATCACTCAGACGCTCCCGGTCACCGTGATTTCATCAAGAACATGATCACTGGTACTTCCTAATCGTCGACCTGCAGGCATGCAAGCTTGGCGTAATCATGGTCATAGCTGTTTCCTGTGTGAAATTGTTATCCGCTCACAATTCCACACAACATACGAGCCGGAAGCATAAAGTGTAAAGCCTGGGGTGCCTAATGAGTGAGCTAACTCACATTAATTGCGTTGCGCTCACTGCCCGCTTTCCAGTCGGGAAACCTGTCGTGCCAGCTGCATTAATGAATCGGCCACCCGCGGGGAAAAGGCGGTTTGCGTATTGGGCGCTCTTCCGCTTCTCCGTTCCTGAATGCTGGGGTCGGGGGTTTGGTGGGGGGAAGGGGTTCATTCCTTCAAGGGGGGGTTAGGGTTTCCCCCAATCAGGGAGTACCCCGGAAAAAATGTGGTCAAAGGCCCCAAGGGCCAGACCTCAAAAAGCGCTTGG

>21SL101 Fusarium oxysporum 100%

GCCTTTGCCCAAAGTTTTTTCCGGGGTTTCCCCGGGTTTGGGGATACCGTATTCCCCCCTTTGAGGACTGATCCCCTTCCCCCAACCGAACGGCCGAGCCCAGGGATCATTAACGAGAAACGGAAGAGCGCCCAATACGCAAACCGCCTTTTCCCCGCGCGTGGCCGATTCATTAATGCAGCTGGCACGACAGGTTTCCCGACTGGAAAGCGGGCAGTGAGCGCAACGCAATTAATGTGAGTTAGCTCACTCATTAGGCACCCCAGGCTTTACACTTTATGCTTCCGGCTCGTATGTTGTGTGGAATTGTGAGCGGATAACAATTTCACACAGGAAACAGCTATGACCATGATTACGCCAAGCTTGCATGCCTGCAGGTCGACGATTATGGGTAAGGAAGACAAGACTCACCTTAACGTCGTCGTCATCGGCCACGTCGACTCTGGCAAGTCGACCACTGTGAGTACTCTCCTCGACAATGAGCATATCTGCCATCGTCAATCCCGACCAAGACCTGGCGGGGTATTTCTCAAAGTCAACATACTGACATCGTTTCACAGACCGGTCACTTGATCTACCAGTGCGGTGGTATCGACAAGCGAACCATCGAGAAGTTCGAGAAGGTTAGTCACTTTCCCTTCAATCGCGCGTCCTTTGCCCATCGATTTCCCCTACGACTCGAAACGTGCCCGCTACCCCGCTCGAGACCAAAAATTTTGCAATATGACCGTAATTTTTTTGGTGGGGCACTTACCCCGCCACTTGAGCGACGGGAGCGTTTGCCCTCTTACCATTCTCAGAACCTCAATGAGTGCGTCGTCACGTGTCAAGCAGTCACTAACCATTCAACAATAGGAAGCCGCTGAGCTCGGTAAGGGTTCCTTCAAGTACGCCTGGGTTCTTGACAAGCTCAAGGCCGAGCGTGAGCGTGGTATCACCATCGATATTGCTCTCTGGAAGTTCGAGACTCCTCGCTACTATGTCACCGTCATTGGTATGTTGTCGCTCATGCTTCATTCTACTTCTCTTCGTACTAACATATCACTCAGACGCTCCCGGTCACCGTGATTTCATCAAGAACATGATCACTGGTACTTCCAATCTCTAGAGGATCCCCGGGTACCGAGCTCGAATTCACTGGCCGTCGTTTTACAACGTCGTGACTGGGAAAACCCTGGCGTTACCCAACTTAATCGCCTTGCAGCACATCCCCCTTTCGCCAGCTGGCGTAATAGCGAAGAGGCCCGCACCGATCGCCCTTCCAACAGTTGCGCAGCCTGAATGGCGAATGGCGCCTGATGCGGGATTTCTCCTTACGCATCTGTGCGGGATTTCACACCGCATATGGGGGACTCTCAGAAAAACTGGTCTGAGGCGCATAATTAAACCACCCCGAAACCGCCAAACCCGGTGAGGGCCTTGAGGGGTTGTTGGTCCCGGTCCCTTTAAAAAAATTGGACGTCCCGGAAGAGGTTTGCA

>21SL108 Fusarium oxysporum 100%

GAAAACCCCACAAGGGGCCTTTTTAGGTTTCGGCCTTTGGGGCCTTTGGCCCCAATTTTTTCCGGGTTATCCCCGGATTTGGGGATACCGTATTCCCCCTTTGAGGAACTGATCCCCTCGCCGCAACCGAACGACCGAGCCCAGCGAGTCAGTGAGGAGGAAACGGAAGAACGCCCAATACGCAAACCGCCTCTCCCCCGCGCGTGGCCGATTCATTAATGCAGCTGGCACGACAGGTTTCCCGACTGGAAAGCGGGCAGTGAGCGCAACGCAATTAATGTGAGTTAGCTCACTCATTAGGCACCCCAGGCTTTACACTTTATGCTTCCGGCTCGTATGTTGTGTGGAATTGTGAGCGGATAACAATTTCACACAGGAAACAGCTATGACCATGATTACGCCAAGCTTGCATGCCTGCAGGTCGACGATTATGGGTAAGGAAGACAAGACTCACCTTAACGTCGTCGTCATCGGCCACGTCGACTCTGGCAAGTCGACCACTGTGAGTACTCTCCTCGACAATGAGCTTATCTGCCATCGTCAATCCCGACCAAGACCTGGCGGGGTACTTCTCAAAGGCAACATACTGACATCGTTTCACAGACCGGTCACTTGATCTACCAGTGCGGTGGTATCGACAAGCGAACCATCGAGAAGTTCGAGAAGGTTAGTCACTTTCCCTTCGATCGCGCGTCCTTTGCCCATCGACTTCCCCTACGACTCGAAACGTGCCCGCTACCCCGCTCGAGACCAAAAATTTTGCAATATGACCGTAATTTTTTTGGTGGGGCATTTACTACGCCACTTGAGCGACGGGGCGCGTTTGCCCTCTTACCATTACAACCTCAATGAGTGCGTCGTCACGTGTCAAGCAGTCACTAACCATTCAACAATAGGAAGCCGCTGAGCTCGGTAAGGGTTCCTTCAAGTACGCCTGGGTTCTTGACAAGCTCAAGGCCGAGCGTGAGCGTGGTATCACCATCGATATTGCTCTCTGGAAGTTCGAGACTCCTCGCTACTATGTCACCGTCATTGGTATGTTGTCGCTCATGCTTCATTCTACTTCTCTTCGTACTAATATATCACTCAGACGCTCCCGGTCACCGTGATTTCATCAAGAACATGATCACTGGTACTTCCAATCTCTAGAGGATCCCCGGGTACCGAGCTCGAATTCACTGGCCGTCGTTTTACAACGTCGTGACTGGGAAAACCCTGGCGTTACCCAACTTAATCGCCTTGCAGCACATCCCCCTTTCGCCAGCTGGCGTAATAGCGAAGAGGCCCGCACCGATCGCCCTTCCCAACAGTTGCGCAGCCTGAATGGCGAATGGCGCCTGATGCGGTATTTTCTCCTTACGCATCTGTGCGGGATTTCACACCGCATATGGTGGACTCTCAGTACAATCTGGTCTGATGCCGCATAGTTAAGCCAGCCCCGAACCCCGCCAAACCCCGGTGAAGGGCCCTGGAGGGGTTTGTTGGTCCCGGAATCCGTTTAG

>21SL113 Fusarium oxysporum 100%

AGCCTACCCTATATATGGGTCCCGGGCCCTTGTTTCGCGTTCGGGAGACGGGAAACTCTGACCCAACAGTCCCGGAGACGTCCCACTTGTTTAAGCGATCCGGGACCAACAACCCCTCAGGCGCGTCAGCGGTGTGGCGGGTTCGGGCTGGCTAACTATGGGCATCAGAGCAGATTGTCTGAGAGTCCCCATATGGGTGTGAATTCCGCACAGATGCGTAGGAGAAAATTCCGCATCAGGCGCCATTCGCCATTCAGGCTGCGCAACTGTTGGGAAGGGCGATCGGTGCGGGCCTCTTCGCTATTACGCCAGCTGGCGAAAGGGGGATGTGCTGCAAGGCGATTAAGTTGGGTAACGCCAGGGTTTTCCCAGTCACGACGTTGTAAAACGACGGCCAGTGAATTCGAGCTCGGTACCCGGGGATCCTCTAGAGATTGGAAGTACCAGTGATCATGTTCTTGATGAAATCACGGTGACCGGGAGCGTCTGAGTGATATGTTAGTACGAAGAGAAGTAGAATGAAGCATGAGCGACAACATACCAATGACGGTGACATAGTAGCGAGGAGTCTCGAACTTCCAGAGAGCAATATCGATGGTGATACCACGCTCACGCTCGGCCTTGAGCTTGTCAAGAACCCAGGCGTACTTGAAGGAACCCTTACCGAGCTCAGCGGCTTCCTATTGTTGAATGGTTAGTGACTGCTTGACACGTGACGACGCACTCATTGAGGTTCTGAGAATGGTAAGAGGGCAAACGCTCCCGTCGCTCAAGTGGCGGGGTAAGTGCCCCACCAAAAAAATTACGGTCATATTGCAAAATTTTTGGTCTCGAGCGGGGTAGCGGGCACGTTTCGAGTCGTAGGGGAAATCGATGGGCAAAGGACGCGCGATTGAAGGGAAAGTGACTAACCTTCTCGAACTTCTCGATGGTTCGCTTGTCGATACCACCGCACTGGTAGATCAAGTGACCGGTCTGTGAAACGATGTCAGTATGTTGACTTTGAGAAATACCCCGCCAGGTCTTGGTCGGGATTGACGATGGCAGATATGCTCATTGTCGAGGAGAGTACTCACAGTGGTCGACTTGCCAGAGTCGACGTGGCCGATGACGACGACGTTAAGGTGAGTCTTGTCTTCCTTACCCAATCGTCGACCTGCAGGCATGCAAGCTTGGCGTAATCATGGTCATAGCTGTTTCCTGTGTGAAATTGTTATCCGCTCACAATTCCACACAACATACGAGCCGGAAGCATAAAGTGTAAAGCCTGGGGTGCCTAATGAGTGAGCTAACTCACATTAATTGCGTTGCGCTCACTGCCCGCTTTCCAGTCGGGAAACCTGTCGTGCCAGCTGCATTAATGAATCGGCCAACGCGCGGGGAAAGGCGGTTTGCGTATTGGGCGCTCTTCCGCTTCCTCGCTCACTGACTCGCTGCGCTCGGTCGTTCGGCTGCGGGAAGGGGATCAGCTCACTCAAAGGCGGGAATACGGTTATCCACAGAATCCGGGGATACCGCCGGAAAGAACTGTGGACCAAAGGCCCCAAAAGGCCCGAACCGTAAAAGGCCC

>21CX122 Fusarium oxysporum 99.86%

GGAAGTACCAGTGATCATGTTCTTGATGAAATCACGGTGACCGGGAGCGTCTGAGTGATATGTTAGTACGAAGAGAAGTAGAATGAAGCATGAGCGACAACATACCAATGACGGTGACATAGTAGCGAGGAGTCTCGAACTTCCAGAGAGCAATATCGATGGTGATACCACGCTCACGCTCGGCCTTGAGCTTGTCAAGAACCCAGGCGTACTTGAAGGAACCCTTACCGAGCTCAGCGGCTTCCTATTGTTGAATGGTTAGTGACTGCTTGACACGTGACGACGCACTCATTGAGGTTCTGAGAATGGTAAGAGGGCAAACGCTCCCGTCGCTCAAGTGGCGGGGTAAGTGCCCCACCAAGAAAATTACGGTCATATTGCAAAATTTTTGGTCTCGAGCGGGGTAGCGGGCACGTTTCGAGTCGTAGGGGAAATCGATGGGCAAAGGACGCGCGATTGAAGGGAAAGTGACTAACCTTCTCGAACTTCTCGATGGTTCGCTTGTCGATACCACCGCACTGGTAGATCAAGTGACCGGTCTGTGAAACGATGTCAGTATGTTGACTTTGAGAAATACCCCGCCAGGTCTTGGTCGGGATTGACGATGGCAGATATGCTCATTGTCGAGGAGAGTACTCACAGTGGTCGACTTGCCAGAGTCGACGTGGCCGATGACGACGACGTTAAGGTGAGTCTTGTCTTCCTTACCCAT

>21SP127 Fusarium oxysporum 99.72%

TGAGCTTATCCCCTTCCCCCCAACCGAACGCCCGAGCCCAGCGATTCAGTGAGCGAGGAAGCGGAAGAGCGCCCAATACGCAAACCGCCTCTTCCCCGCGCGTTGGCCGATTCATTAATGCAGCTGGCACGACAGGTTTCCCGACTGGAAAGCGGGCAGTGAGCGCAACGCAATTAATGTGAGTTAGCTCACTCATTAGGCACCCCAGGCTTTACACTTTATGCTTCCGGCTCGTATGTTGTGTGGAATTGTGAGCGGATAACAATTTCACACAGGAAACAGCTATGACCATGATTACGCCAAGCTTGCATGCCTGCAGGTCGACGATTATGGGTAAGGAAGACAAGACTCACCTTAACGTCGTCGTCATCGGCCACGTCGACTCTGGCAAGTCGACCACTGTGAGTACTCTCCTCGACAATGAGCTTATCTGCCATCGTCAATCCCGACCAAGACCTGGCGGGGTACTTCTCAAAGGCAACATACTGACATCGTTTCACAGACCGGTCACTTGATCTACCAGTGCGGTGGTATCGACAAGCGAACCATCGAGAAGTTCGAGAAGGTTAGTCACTTTCCCTTCGATCGCGCGTCCTTTGCCCATCGACTTCCCCTACGACTCGAAACGTGCCCGCTACCCCGCTCGAGACCAAAAATTTTGCAATATGACCGTAATTTTTTTGGTGGGGCATTTACCCCGCCACTTGAGCGACGGGGCGCGTTTGCCCTCTTACCATTACAACCTCAATGAGTGCGTCGTCACGTGTCAAGCAGTCACTAACCATTCAACAATAGGGAGCCGCTGAGCTCGGTAAGGGTTCCTTCAAGTACGCCTGGGTTCTTGACAAGCTCAAGGCCGAGCGTGAGCGTGGTATCACCATCGATATTGCTCTCTGGAAGTTCGAGACTCCTCGCTACTATGTCACCGTCATTGGTATGTTGTCGCTCATGCTTCATTCTACTTCTCTTCGTACTAATATATCACTCAGACGCTCCCGGTCACCGTGATTTCATCAAGAACATGATCACTGGTACTTCCAATCTCTAGAGGATCCCCGGGTACCGAGCTCGAATTCACTGGCCGTCGTTTTACAACGTCGTGACTGGGAAAACCCTGGCGTTACCCAACTTAATCGCCTTGCAGCACATCCCCCTTTCGCCAGCTGGCGTAATAGCGAAGAGGCCCGCACCGATCGCCCTTCCCAACAGTTGCGCAGCCTGAATGGCGAATGGCGCCTGATGCGGTATTTTCTCCTTACGCATCTGTGCGGTATTTCACACCGCATATGGGGGACTCTCAGTACAATCTGGTCTGAAGCCGCATAATTAAACCAGCCCCGAAACCCGC

>21SP129 Fusarium oxysporum 100%

CTGGGGATACCCGTATCCCGCCCTTGGAGGAGGTGATCCCTTTCCCCCAACCGAACGCCCGAGCCCACCGATCCATTGGCGAGGAACCGGAAGAGCGCCCAATACGCAAACCGCCTTTTCCCCGCGCGTTGGCCGATTCATTAATGCAGCTGGCACGACAGGTTTCCCGACTGGAAAGCGGGCAGTGAGCGCAACGCAATTAATGTGAGTTAGCTCACTCATTAGGCACCCCAGGCTTTACACTTTATGCTTCCGGCTCGTATGTTGTGTGGAATTGTGAGCGGATAACAATTTCACACAGGAAACAGCTATGACCATGATTACGCCAAGCTTGCATGCCTGCAGGTCGACGATTATGGGTAAGGAAGACAAGACTCACCTTAACGTCGTCGTCATCGGCCACGTCGACTCTGGCAAGTCGACCACTGTGAGTACTCTCCTCGACAATGAGCTTATCTGCCATCGTCAATCCCGACCAAGACCTGGCGGGGTACTTCTCAAAGGCAACATACTGACATCGTTTCACAGACCGGTCACTTGATCTACCAGTGCGGTGGTATCGACAAGCGAACCATCGAGAAGTTCGAGAAGGTTAGTCACTTTCCCTTCGATCGCGCGTCCTTTGCCCATCGACTTCCCCTACGACTCGAAACGTGCCCGCTACCCCGCTCGAGACCAAAAATTTTGCAATATGACCGTAATTTTTTTGGTGGGGCATTTACCCCGCCACTTGAGCGACGGGGCGCGTTTGCCCTCTTACCATTACAACCTCAATGAGTGCGTCGTCACGTGTCAAGCAGTCACTAACCATTCAACAATAGGAAGCCGCTGAGCTCGGTAAGGGTTCCTTCAAGTACGCCTGGGTTCTTGACAAGCTCAAGGCCGAGCGTGAGCGTGGTATCACCATCGATATTGCTCTCTGGAAGTTCGAGACTCCTCGCTACTATGTCACCGTCATTGGTATGTTGTCGCTCATGCTTCATTCTACTTCTCTTCGTACTAATATATCACTCAGACGCTCCCGGTCACCGTGATTTCATCAAGAACATGATCACTGGTACTTCCAATCTCTAGAGGATCCCCGGGTACCGAGCTCGAATTCACTGGCCGTCGTTTTACAACGTCGTGACTGGGAAAACCCTGGCGTTACCCAACTTAATCGCCTTGCAGCACATCCCCCTTTCGCCAGCTGGCGTAATAGCGAAGAGGCCCGCACCGATCGCCCTTCCCAACAGTTGCGCAGCCTGAATGGCGAATGGCGCCTGATGCGGTATTTTCTCCTTACGCATCTGTGCGGGATTTCACACCGCATATGGGGGACTCTCAGTACAATCTGGTCTGATGCCGCATAATTAAAGCAAGCCCGAAACCCGGCAAAACCCGGTAGAGG

>21SP130 Fusarium oxysporum 100%

TTTTTCCCCTTGAGGAACGAACCCCTCCGCCACCCAAGCCCCACCCAGCAATCAGTAGCGAGAACCGAAGACCCCCAAACGCAAACCGCCTTCCCCGCGCGTGGCCGATTCATTAAGCAGCGGCACGACAGGTTCCCGACTGAAAGCGGGCAGGAGCGCAACGCAATTAAGTGAGTTAGCTCACTCATTAGGCACCCCAGGCTTTACACTTTATGCTTCCGGCTCGTATGTTGTGTGGAATTGTGAGCGGATAACAATTCACACAGGAAACAGCTATGACCATGATTACGCCAAGCTTGCATGCCTGCAGGTCGACGATTATGGGTAAGGAAGACAAGACTCACCTTAACGTCGTCGTCATCGGCCACGTCGACTCTGGCAAGTCGACCACTGTGAGTACTCTCCTCGACAATGAGCTTATCTGCCATCGTCAATCCCGACCAAGACCTGGCGGGGTACTTCTCAAAGGCAACATACTGACATCGTTTCACAGACCGGTCACTTGATCTACCAGTGCGGTGGTATCGACAAGCGAACCATCGAGAAGTTCGAGAAGGTTAGTCACTTTCCCTTCGATCGCGCGTCCTTTGCCCATCGACTTCCCCTACGACTCGAAACGTGCCCGCTACCCCGCTCGAGACCAAAAATTTTGCAATATGATCGTAATTTTTTTGGTGGGGCATTTACCCCGCCACTTGAGCGACGGGGCGCGTTTGCCCTCTTACCATTACAACCTCAATGAGTGCGTCGTCACGTGTCAAGCAGTCACTAACCATTCAACAATAGGAAGCCGCTGAGCTCGGTAAGGGTTCCTTCAAGTACGCCTGGGTTCTTGACAAGCTCAAGGCCGAGCGTGAGCGTGGTATCACCATCGATATTGCTCTCTGGAAGTTCGAGACTCCTCGCTACTATGTCACCGTCATTGGTATGTTGTCGCTCATGCTTCATTCTACTTCTCTTCGTACTAATATATCACTCAGACGCTCCCGGTCACCGTGATTTCATCAAGAACATGATCACTGGTACTTCCAATCTCTAGAGGATCCCCGGGTACCGAGCTCGAATTCACTGGCCGTCGTTTTACAACGTCGTGACTGGGAAAACCCTGGCGTTACCCAACTTAATCGCCTTGCAGCACATCCCCCTTTCGCCAGCTGGCGTAATAGCGAAGAGGCCCGCACCGATCGCCCTTCCCAACAGTTGCGCAGCCTGAATGGCGAATGGCGCCTGATGCGGTATTTTCTCCTTACGCATCTGTGCGGGATTTCACACCGCATATGGGGGACTCTCAGTACAATCTGGTCTGAAGGCGCATAATTAAACCAGCCCCCAAACCCGCCAAAACCGGT

>21SP134 Fusarium oxysporum 99.72%

ACGCCCGAGCCCAACGATTCAGTGAGCGAGGAAGCGGAAGAGCGCCCAATACGCAAACCGCTTCTCCCCGCGCGTTGGCCGATTCATTAATGCAGCTGGCACGACAGGTTTCCCGACTGGAAAGCGGGCAGTGAGCGCAACGCAATTAATGTGAGTTAGCTCACTCATTAGGCACCCCAGGCTTTACACTTTATGCTTCCGGCTCGTATGTTGTGTGGAATTGTGAGCGGATAACAATTTCACACAGGAAACAGCTATGACCATGATTACGCCAAGCTTGCATGCCTGCAGGTCGACGATTATGGGTAAGGAAGACAAGACTCACCTTAACGTCGTCGTCATCGGCCACGTCGACTCTGGCAAGTCGACCACTGTGAGTACTCTCCTCGACAATGAGCTTATCTGCCATCGTCAATCCCGACCAAGACCTGGCGGGGTACTTCTCAAAGGCAACATACTGACATCGTTTCACAGACCGGCCACTTGATCTACCAGTGCGGTGGTATCGACAAGCGAACCATCGAGAAGTTCGAGAAGGTTAGTCACTTTCCCTTCGATCGCGCGTCCTTTGCCCATCGACTTCCCCTACGACTCGAAACGTGCCCGCTACCCCGCTCGAGACCAAAAATTTTGCAATATGACCGTAATTTTTTTGGTGGGGCATTTACCCCGCCACTTGAGCGACGGGGCGCGTTTGCCCTCTTACCATTACAACCTTAATGAGTGCGTCGTCACGTGTCAAGCAGTCACTAACCATTCAACAATAGGAAGCCGCTGAGCTCGGTAAGGGTTCCTTCAAGTACGCCTGGGTTCTTGACAAGCTCAAGGCCGAGCGTGAGCGTGGTATCACCATCGATATTGCTCTCTGGAAGTTCGAGACTCCTCGCTACTATGTCACCGTCATTGGTATGTTGTCGCTCATGCTTCATTCTACTTCTCTTCGTACTAATATATCACTCAGACGCTCCCGGTCACCGTGATTTCATCAAGAACATGATCACTGGTACTTCCAATCTCTAGAGGATCCCCGGGTACCGAGCTCGAATTCACTGGCCGTCGTTTTACAACGTCGTGACTGGGAAAACCCTGGCGTTACCCAACTTAATCGCCTTGCAGCACATCCCCCTTTCGCCAGCTGGCGTAATAGCGAAGAGGCCCGCACCGATCGCCCTTCCCAACAGTTGCGCAGCCTGAATGGCGAATGGCGCCTGATGCGGTATTTTCTCCTTACGCATCTGTGCGGTATTTCACACCGCATATGGGGCACTCTCAGAACAATCTGGTCTGATGCCGCAAATTAAGCCAGCCCGAAACCC

>21ES143 Fusarium oxysporum 100%

GGAAGTACCAGTGATCATGTTCTTGATGAAATCACGGTGACCGGGAGCGTCTGGGTGATATGTTAGTACGAAGAGAAGTAGAATGAAGCATGAGCGACAACATACCAATGACGGTGACATAGTAGCGAGGAGTCTCGAACTTCCAGAGAGCAATATCGATGGTGATACCACGCTCACGCTCGGCCTTGAGCTTGTCAAGAACCCAGGCGTACTTGAAGGAACCCTTACCGAGCTCAGCGGCTTCCTATTGTTGAGTGGTTAGTGACTGCTTGACACGTGACGACGCACTCATTGAGGTTGTGAGAATGGTAAGAGGGCAAACGCTCCCGTCGCTCAAGTGGCGGGGTAAGTGCCCCACCAAAAAAATTACGGTCATATTGCAAAATTTTTGGTCTCGAGCGGGGTAGCGGGCACGTTTCGAGTCGTAGGGGAAATCGATGGGCAAAGGACGCGCGATTGAAGGGAAAGTGACTAACCTTCTCGAACTTCTCGATGGTTCGCTTGTCGATACCACCGCACTGGTAGATCAAGTGACCGGTCTGTGAAACGATGTCAGTATGTTGACTTTGAGAAATACCCCGCCAGGTCTTGGTCGGGATTGACGATGGCAGATATGCTCATTGTCGAGGAGAGTACTCACAGTGGTCGACTTGCCAGAGTCGACGTGGCCGATGACGACGACGTTAAGGTGAGTCTTGTCTTCCTTACCCAT

>21ES145 Fusarium oxysporum 99.72%

GGAAGTACCAGTGATCATGTTCTTGATGAAATCACGGTGACCGGGAGCGTCTGGGTGATATGTTAGTACGAAGAGAAGTAGAATGAAGCATGAGCGACAACATACCAATGACGGTGACATAGTAGCGAGGAGTCTCGAACTTCCAGAGAGCAATATCGATGGTGATACCACGCTCACGCTCGGCCTTGAGCTTGTCAAGAACCCAGGCGTACTTGAAGGAACCCTTACCGAGCTCAGCGGCTTCCTATTGTTGAGTGGTTAGTGACTGCTTGACACGTGACGACGCACTCATTGAGGTTGTGAGAATGGTAAGAGGGCAAACGCTCCCGTCGCTCAAGTGGCGGGGTAAGTGCCCCACCAAAAAAATTACGGTCATATTGCAAAATTTTTGGTCTCGAGCGGGGTAGCGGGCACGTTTCGAGTCGTAGGGGAAATCGATGGGCAAAGGACGCGCGATTGAAGGGAAAGTGACTAACCTTCTCGAACTTCTCGATGGTTCGCTTGTCGATACCACCGCACTGGTAGATCAAGTGACCGGTCTGTGAAACGATGTCAGTATGTTGACTTTGAGAAATACCCCGCCAGGTCTTGGTCGGGATTGACGATGGCAGATATGCTCATTGTCGAGGAGAGTACTCACAGTGGTCGACTTACCAGAGTCGACGTGGCCGATGACGACGACGTTAAGGTGAGTCTTGTCTTCCTTACCCAT
